# Factors Associated with Infectious Diseases Fellowship Academic Success

**DOI:** 10.21203/rs.3.rs-3140095/v1

**Published:** 2023-07-28

**Authors:** Ryan B. Khodadadi, Zachary A. Yetmar, Cynthia L. Domonoske, Raymund R. Razonable

**Affiliations:** Mayo Clinic; Mayo Clinic; Mayo Clinic; Mayo Clinic

**Keywords:** Infectious diseases fellowship, academic susccess, medical educaiton, outcomes

## Abstract

**Background:**

A multitude of factors are considered in an infectious diseases (ID) training program’s meticulous selection process of ID fellows but their correlation to pre and in-fellowship academic success as well as post-fellowship academic success and short-term outcomes is poorly understood. Our goal was to investigate factors associated with subsequent academic success in fellowship as well as post-fellowship short-term outcomes.

**Methods:**

In 2022, we retrospectively analyzed deidentified academic records from 39 graduates of the Mayo Clinic Rochester ID Fellowship Program (July 1, 2013- June 30, 2022). Data abstracted included demographics, degrees, honor society membership, visa/citizenship status, medical school, residency training program, United States Medical Licensure Exam (USMLE) scores, letters of recommendation, in-training examination (ITE) scores, fellowship track, academic rank, career choice, number of honors, awards, and abstracts/publications prior to fellowship, during training, and within 2 years of graduation.

**Results:**

Younger fellows had higher USMLE step 1 scores, pre and in-fellowship scholarly productivity, and higher ITE performance. Female fellows had significantly higher USMLE step 3 scores. Prior research experience translated to greater in-fellowship scholarly productivity. Higher USMLE scores were associated with higher ID ITE performance during multiple years of fellowship, but USMLE step 2 clinical knowledge and 3 scores were associated with higher pre and in-fellowship scholarly productivity and receiving an award during fellowship. USMLE step 1 score did not correlate with fellowship performance beyond year 1 and 2 ITE scores.

**Conclusions:**

Multiple aspects of a prospective fellow’s application must be considered as part of a holistic reviewprocess for fellowship selection. USMLE step 2 CK and 3 scores may predict fellowship performance across multiple domains.

## Background

Dedicated subspecialty training in infectious diseases (ID) is designed to provide a comprehensive experience in clinical practice, research, and education to allow graduates to practice independently in a variety of settings including academic and community medical centers. Fellowship positions are filled through the National Resident Matching Program (NRMP), in an “all-in” match on a yearly basis. The application, interview, and ranking process is similar in fashion to prerequisite internal medicine (IM) residency training.

As part of this process, ID fellowship selection committees are tasked with comprehensively reviewing multiple aspects of a prospective fellow’s application including pre-medical, medical school and IM residency performance, United States Medical Licensing Examination (USMLE) step 1, 2 clinical knowledge (CK), and 3 scores, program director and faculty letters of recommendation, and scholarly activity. Following this rigorous initial holistic review, candidates are selected to interview for available positions through the NRMP match. At the conclusion of the interview process, programs’ rank order lists of applicants is created after taking into consideration pre-interview application strength, interview interactions with program director and selection committee members, faculty, staff, and current trainees as well as institutional fit. This list is then submitted to the NRMP to facilitate the match process.

Despite this approach to the review of prospective fellows’ applications, selection of interview candidates and subsequent creation of a program rank order list, the factors taken into consideration in the selection and ranking of applicants and their association with academic success during and after ID fellowship are not well characterized. Factors associated with fellowship academic success have been identified in previous studies of larger IM subspecialties, but not for ID fellowships to our knowledge^1, 2^. The comparatively lower number of ID fellows relative to other IM subspecialty training programs such as gastroenterology, cardiology, and hematology/oncology have limited the feasibility of such a study. Additionally, this is compounded by historically lower fill rates of ID fellowship programs over the years^3–5^.

Given the paucity of data surrounding factors associated with fellowship academic success in the current literature, we sought to perform a retrospective review of trainees to identify factors associated with academic success among ID fellows in our program.

## Methods

The Mayo Clinic School of Graduate Medical Education (MCSGME) ID Fellowship Program, founded in 1961 in Rochester, Minnesota, is a three-year training program which provides a robust and immersive clinical experience for trainees with protected research time, guaranteed institutional-based funding for research, and a variety of subspecialty ID tracks to individualize training according to the trainee’s career goals through multiple individualized fellowship tracks. Beginning in their second year, ID fellows are eligible for nomination to the Academic Appointment and Promotions Committee for instructor and assistant professor of medicine academic ranks, depending on their scholarship and other measures. The program has a 100% American Board of Internal Medicine (ABIM) ID Certification Exam pass rate. Currently, the program recruits fellows to fill 6 available positions each year through the fellowship recruitment and NRMP match process.

Following Mayo Clinic Institutional Review Board approval, we performed a retrospective cohort study of graduates of the Mayo Clinic ID fellowship program over a decade-long period between 2013–2022 by reviewing deidentified trainee academic records. During this period, the number of fellows increased from 3 per year to 6 per year. Prior to data collection and analysis, the study protocol was reviewed by the Educational Research Committee of the Mayo Clinic Institutional Review Board and found to be in compliance with the learners as research subjects policy at Mayo Clinic. Data abstracted included demographics, medical degree, additional advanced degrees, honor society membership, visa/citizenship status, medical school, residency training program type and size, USMLE step 1, 2CK, and 3 scores, letter of recommendation with note of a top qualifier, in-training examination (ITE) scores, fellowship training track, academic rank at graduation, career choice, number of abstracts, honors, awards, and publications prior to matriculation into fellowship, during training and within 2 years of graduation.

Data in the accompanying tables are displayed as either frequency (%) for nominal variables or median (range) for continuous variables. Spearman’s correlations were used to test associations between two continuous variables. Associations between continuous predictors and binary outcomes were tested by logistic regression. Two-sided t tests or analysis of variance models were used to test associations between binary or multinominal predictors, respectively, and continuous outcomes. Fisher’s exact test was used to test associations between two binary variables. All analyses were performed on RStudio statistical software, version 2022.07.1.554 (RStudio Team, PBC, Boston, Massachusetts, United States).

## Results

Over the study time frame, there were thirty-nine ID fellowship program graduates with the accompanying characteristics. Twenty-one fellows were male (53.8%) and the median age at the beginning of ID fellowship was 31 years (range 26–42). Sixteen (41%) of trainees were United States citizens, 35.9% had a J1 visa, 20.5% had a H1b visa and one fellow (2.6%) was a permanent United States resident.

Prior to beginning ID fellowship, twenty-five fellows (64.1%) were international medical graduates. Median USMLE step 1, 2 CK, and 3 scores were 240, 246, and 233, respectively. Twenty-three fellows (59%) completed IM residency training at an academic medical center and 33% of trainees attended a residency program with more than 50 trainees per residency class. Five fellows (12.8%) completed additional training as a Chief Medical Resident. The median number of abstracts and publications prior to fellowship were 5 and 3, respectively.

During training, the median number of abstracts was 6 (range, 1–21) and the median number of publications was 6 (range, 0–54). The most common fellowship tracks were the traditional clinician scholar followed by intensive care (paired with antimicrobial stewardship and infection control), transplant and immunocompromised host, and research tracks. Thirty-six (92.6%) of fellows received an award during training and 97.4% of graduates took ID consultant positions at academic institutions. Additional cohort characteristics are available in Table 1.

### Factors associated with pre-fellowship performance

Female gender was associated with a higher USMLE step 3 score (p = .041), while a younger age was associated with a higher USMLE step 1 score and a higher number of pre-fellowship publications (p = .021 and .032, respectively). Further, a higher USMLE step 2 score was associated with a higher number of pre-fellowship publications (p = .031). The results of this analysis of the effect of demographic factors and pre-fellowship variables are shown in Table 2.

### Factors associated with performance during ID fellowship

The results of the analysis of associations between pre-fellowship and in-fellowship variables are displayed in Table 3. A younger age was found to be associated with higher year 1 and year 2 ITE scores (p = .027 and .025, respectively) and higher number of publications during fellowship (p = .005). Graduating from an academic/university-based residency program was associated with higher likelihood of receiving the Geraci award, given to one graduating fellow each year in recognition of excellent performance in clinical care, education, and research during ID fellowship (p = .037), while a higher number of pre-fellowship abstracts was associated with higher numbers of abstracts and publications during fellowship (p = .026 and .002 respectively).

A higher USMLE step 1 score was associated with higher year 1 and year 2 ITE scores whereas a higher USMLE step 2 score was associated with higher ITE scores across all 3 years and higher likelihood to receive the Geraci award (Table 3). Additionally, USMLE step 3 performance was associated with higher year 1 and year 2 ITE scores, a higher number of publications during fellowship, higher likelihood of receiving an award during fellowship, and higher likelihood to receive the Geraci award (Table 3).

### Factors associated with post-fellowship outcomes

With regards to the association of in-fellowship variables and outcomes data, fellows who received an award during fellowship had higher numbers of publications both during fellowship and during their first two years after fellowship graduation (p = < .001 and .005, respectively). Post-hoc pairwise analysis showed those appointed to the academic rank of Assistant Professor of Medicine had higher numbers of abstracts and publications during fellowship as compared to those appointed to instructor of medicine rank (Table 4). In addition, years 1–3 ITE performance as well as the receipt of the Geraci award were associated with a higher score on the ABIM ID Certification Exam (Table 4).

## Discussion

The benefit of ID subspecialty evaluation has previously been highlighted through studies demonstrating improved survival and outcomes in patients with blood stream infections and drug-resistant organisms^6–8^. The importance of ID expertise in routine clinical care and ongoing public health efforts has been further accentuated throughout the COVID-19 pandemic and moving forward, the need for subspecialty training in ID continues to be of the utmost importance for ongoing pandemic preparedness^9^.

Prior studies have characterized factors associated with academic success and career choice within hematology and oncology fellowship training^1^. Ours is the first study of its kind to identify factors associated with ID fellowship academic success, taking into consideration data provided during the fellowship application process and the association on subsequent fellowship achievement and academic success. Several associations were observed among demographic, pre-fellowship, in-fellowship, and post-fellowship variables from our analysis of the Mayo Clinic Rochester ID Fellowship Program trainees from 2013–2022.

Among demographic characteristics, younger fellows have significantly higher USMLE step 1 score, pre-and in-fellowship scholarly productivity, and ITE performance. Female fellows had significantly higher USMLE step 3 scores.

Several pre-fellowship variables were significantly correlated with academic performance during ID fellowship training. Having a prior research experience translated to significantly higher in-fellowship scholarly productivity. There was also association among scores on pre-fellowship standardized examinations (USMLE) and subsequent performance in-fellowship examinations (ITE). In our study, higher USMLE scores were associated with higher performance on the ID ITE during multiple years of fellowship, confirming findings from prior studies demonstrating this association^10^. Beyond their association with ITE performance, higher USMLE step 2 CK and 3 scores were also associated with higher pre and in-fellowship scholarly productivity as well as likelihood of receiving an award during fellowship. Interestingly, in contrast, USMLE step 1 score did not significantly correlate with fellowship performance beyond the year 1 and 2 ITE scores. Similar correlations of USMLE examination performance to ID ITE equivalents have been observed across other non-IM specialties including orthopedic surgery, plastic surgery, anesthesiology, emergency medicine, and obstetrics and gynecology residencies^11–15^.

Applications of this work include its potential to guide both ID fellowship selection committees and prospective applicants through subsequent application cycles^16^. The association between pre-fellowship research experience and in-fellowship research productivity may be useful for programs geared towards training academic clinician-scientists. The association between USMLE step 2 CK and 3 performance and in-fellowship success variables across multiple domains suggests that these examinations may serve as additional tools for selection committees when considering a candidate’s potential for in-fellowship academic success. The importance of the USMLE step 2 CK and 3 examinations may be further underscored in future application cycles as the USMLE step 1 recently transitioned to a pass/fail examination in 2022. While unique to our study, previous research outside of ID to evaluate the utility of USMLE step 2 CK on prediction of training performance has been mixed. This examination has previously been shown to be a predictor of multimodal performance during IM residency in the ambulatory setting whereas this examination was not found to be a reliable predictor American Board of Neurological Surgery written exam performance^17, 18^.

Limitations of this study include its generalizability across ID fellowship programs. First, this retrospective study was conducted at a large academic medical center in the Midwestern United States. Secondly, the Mayo Clinic Rochester ID Fellowship Program is a three-year program for all its general ID fellows, providing them with the opportunity for academic advancement. Our results may therefore reflect programs of the same length of training. Admittedly, this is not the same structure for most ID fellowship programs in the country, where 2 years is the standard duration of training for eligibility for certification by the ABIM. Accordingly, measures of scholarly productivity in this study may not be applicable to most two-year clinical training programs. However, there are other findings that may be pertinent to all programs, such as the strong association among standardized examinations, and other factors that predict excellence in clinical skills, research, and education (as measured by the Geraci award in this program). Future investigations may be targeted towards elucidating the effect of pre and in-fellowship variables on in-fellowship clinical performance as determined through review of standardized evaluations completed during fellowship training mapped to the Accreditation Council for Graduate Medical Education (ACGME) Infectious Disease Milestones.

## Conclusions

In conclusion, this study of a relatively large academic training program in ID observed several variables that predict performance during fellowship training and short-term outcomes after graduation from the ID fellowship training program.

## Figures and Tables

**Table 1:** Characteristics of 39 infectious diseases fellows^,2^

Variable	Overall (N = 39)
Sex	
- Female	18 (46.2)
- Male	21 (53.8)
Age at start of fellowship, years, median (range)	31 (26–42)
Medical degree	
- M.D.	26 (66.7)
- M.B.B.S.	11 (28.2)
- M.B., B.Ch., B.A.O.	1 (2.6)
- M.B., Ch.B.	1 (2.6)
Ph.D.	2 (5.1)
Medical school location	
- International	25 (64.1)
- United States	14 (35.9)
Visa resident citizenship status	
- US citizen	16 (41.0)
- Permanent US resident	1 (2.6)
- J1 visa	14 (35.9)
- H1B visa	8 (20.5)
Alpha Omega Alpha status (N = 38)	4 (10.5)
Gold Humanism Honor Society status (N = 38)	1 (2.6)
USMLE Step 1, median (range)	240 (198–269)
USMLE Step 2 clinical knowledge, median (range)	246 (216–275)
USMLE Step 3, median (range) (N = 38)	233 (197–253)
Residency program type	
- Academic/university	23 (59.0)
- Community	16 (41.0)
Residency program size, number of residents per year	
- < 15	9 (23.1)
- 15–24	7 (17.9)
- 25–34	6 (15.4)
- 35–44	4 (10.3)
- > 50	13 (33.3)
Chief medical resident	5 (12.8)
Clinical investigator	3 (7.7)
Letter of recommendation with top qualifier (N = 38)	4 (10.5)
Abstracts pre-fellowship, median (range)	5 (0–14)
Publications pre-fellowship, median (range)	3 (0–18)
Year 1 Infectious diseases ITE, median (range) (N = 38)	512 (359–727)
Year 2 Infectious diseases ITE, median (range)	573 (455–800)
Year 3 Infectious diseases ITE, median (range) (N = 31)	586 (436–800)
Infectious diseases board examination score, median (range) (N = 28)	591.5 (393–800)
Publications during fellowship, median (range)	6 (0–54)
Abstracts during fellowship, median (range) (N = 38)	6 (1–21)
Award during fellowship	36 (92.3)
- Geraci award	13 (33.3)
- Divisional	22 (56.4)
- Institutional	9 (23.1)
- National	32 (82.1)
Awards during fellowship, median (range)	3 (0–7)
Chief fellow designation	15 (38.5)
Fellowship track	
- Intensive care	9 (23.1)
- Orthopedic	2 (5.1)
- Research	9 (23.1)
- Traditional clinician scholar	10 (25.6)
- Transplant/immunocompromised host	9 (23.1)
Academic appointment at graduation	
- Assistant professor of medicine	22 (56.4)
- Instructor of medicine	14 (35.9)
- None	3 (7.7)
Career Choice	
- Academic	38 (97.4)
- Private practice	1 (2.6)
Publications within 2 years post-fellowship, median (range)	4 (0–26)

Abbreviations: ITE, In-Training Examination; US, United States; USMLE, United States Medical Licensing Examination

2 Data are N (%) unless otherwise specified.

**Table 2:** Associations between demographic factors and pre-fellowship variables^1,2^

Pre-fellowship variable						
Demographic variable	USMLE 1	USMLE 2 CK	USMLE 3	LOR top qualifier	Abstracts pre-fellowship	Publications pre-fellowship
Sex	0.271	0.283	**0.041**	0.606	0.978	0.520
Age	**0.021**	0.223	0.298	0.969	0.066	**0.032**
Citizenship status	0.911	0.666	0.759	0.841	0.959	0.388
Med school location	0.899	0.512	0.649	0.278	0.280	0.247

Early pre-fellowship variable	Late pre-fellowship variable		
	LOR top qualifier	Abstracts pre-fellowship	Publications pre-fellowship
USMLE 1	0.320	0.256	0.483
USMLE 2 CK	0.581	0.258	**0.031**
USMLE 3	0.395	0.246	0.849

Abbreviations: ITE, In-Training Examination; US, United States; USMLE, United States Medical Licensing Examination; CK, Clinical Knowledge; LOR, Letter of Recommendation

2 Data are N (%) unless otherwise specified.

**Table 3:** Associations between pre-fellowship and in-fellowship variables^1,2^

Pre-fellowship variable	In-fellowship variable						
	Year 1 ITE	Year 2 ITE	Year 3 ITE	Abstracts during fellowship	Publications during fellowship	Award during fellowship	Geraci award
Sex	0.590	0.688	0.540	0.322	0.654	1.000	1.000
Age	**0.027**	**0.025**	0.789	0.091	**0.005**	0.200	0.164
Citizenship status	0.298	0.296	0.811	0.723	0.521	0.215	1.000
Medical school location	0.745	0.236	0.773	0.185	0.413	1.000	0.482
USMLE 1	**0.002**	**0.041**	0.069	0.523	0.709	0.497	0.350
USMLE 2 CK	**0.003**	**0.010**	**0.003**	0.456	0.454	0.114	**0.015**
USMLE 3	**0.008**	**0.045**	0.051	0.454	**0.042**	**0.046**	**0.022**
LOR top qualifier	0.652	0.388	0.454	0.792	0.518	1.000	0.730
Residency program type	0.161	0.796	0.986	0.666	0.282	0.557	**0.037**
Residency program size	0.940	0.161	0.759	0.166	0.782	1.000	0.863
Chief resident	0.195	0.374	0.332	0.803	0.422	1.000	1.000
Abstracts pre-fellowship	0.570	0.135	0.990	**0.026**	**0.002**	0.428	0.910
Publications pre-fellowship	0.901	0.973	0.968	0.061	0.088	0.658	0.683

Abbreviations: ITE, In-Training Examination; US, United States; USMLE, United States Medical Licensing Examination; CK, Clinical Knowledge; LOR, Letter of Recommendation

2 Data are N (%) unless otherwise specified.

**Table 4:** Associations between in-fellowship variables and outcomes^1,2^

In-fellowship quantitative	Research productivity			
	Abstracts during fellowship	Publications during fellowship	Publications within 2 years post-fellowship	Infectious diseases board examination score
Year 1 ITE	0.825	0.327	0.215	**0.002**
Year 2 ITE	0.479	0.120	0.638	**<0.001**
Year 3 ITE	0.465	0.417	0.445	**0.003**
Chief fellow	0.145	0.229	0.578	0.523
Fellowship track	0.100	0.069	0.822	0.712
Award during fellowship	0.288	**<0.001**	**0.005**	0.903
Geraci award	0.263	0.967	0.755	**0.044**
Academic appointment	**0.008**	**0.017**	0.393	0.537

Abbreviations: ITE, In-Training Examination

2 Data are N (%) unless otherwise specified.

## Data Availability

The datasets used and/or analysed during the current study are available from the corresponding author on reasonable request.

## Abbreviations

ABIM: American Board of Internal Medicine
CK: Clinical Knowledge
ID: Infectious Diseases
IM: Internal Medicine
ITE: In-Training Examination
MCSGME: Mayo Clinic School of Graduate Medical Education
NRMP: National Resident Matching Program
USMLE: United Stated Medical Licensing Examination
